# Early infantile epileptic encephalopathies

**DOI:** 10.1186/1824-7288-40-S1-A69

**Published:** 2014-08-11

**Authors:** Alberto Fois

**Affiliations:** 1 Siena, Italy

## 

Epileptiform abnormalities contribute to progressive deterioration of cerebral function. Considered: Ohtahara Syndrome; Early myoclonic epileptic encephalopathy; West Syndrome; Dravet Syndrome; Myoclonic status in not progressive encephalopathies; CDKL5 encephalopaty.

**Ohtahara syndrome (OS)** early infantile encephalopathy (EIEE). Most cases linked to cerebral malformations or very occasionally to metabolic disorders. Main seizures: tonic spasms, tonico- clonic, myoclonic and atonic and partial spasms. Treatment Vigabatrin, Topamax, Zonegran, Steroids, ACTH and Ketogenic diet, vagal stimulation and more invasive surgery. Mutations of the gene STBX1 (Sintaxin binding protein) Mutations of SPTA gene and GC1 have been reported. Prognosis is poor.

**Early Myoclonic Encephalopathy (EME).** Onset: neonatal period or first months of life. Seizures mainly erratic, myoclonic or partial, tonic spasms. EEG burst suppression. Etiology mostly unknown. Inborn errors of metabolism as nonketotic hyperglycinemia, or propionic acidemia reported . Prognosis poor. Therapy similar to that for OS.

**West Syndrome (WS)** aka as infantile spasms with hypsarrhythmia, EEG abnormality with asynchronous very high amplitude, irregular, continuous multifocal spike an slow wave discharges. In 80% of cases a cause can be found or suspected (symptomatic or criptogenethic)In about 20% of patients no cause can be found (idiopathic). 1:3200 / 1:3500 newborns. Causes: infections, cerebral malformations, hypoxic ischemic injuries, metabolic and genetic disorders, tuberosclerosis. Corticotropin considered effective. This finding later confirmed. No definite therapeutic scheme. High dose and long term treatment associated with hypertension, cuschingoid features and ipokalemia. Vigabatrin and Topiramate useful. Zonisamide, Lamotrigine and Levetiracetam can be used. **P**rognosis much better in idiopathic cases when treatment is started within the first month from the appearance of the symptoms.

**Myoclonic status in nonprogressive encephalopathies.** Rare, onset in the first years of life. **P**artial motor seizures, myoclonic absences or massive myoclonias sometimes with startles. Interictal EEG epileptiform discharges and background slowing. Described in genetic conditions like Angelman and 4p – syndromes. Prognosis poor.

**Dravet syndrome (Severe myoclonic infantile epilepsy or SMEI).** 1/500 cases of infantile epilepsy. Febrile convulsions in the first year. Subsequently myoclonic seizure with fever. Photosensitivity. In 70% of cases mutations of SCN1A gene. Difficult to treat. Topiramate, Clobazam, Stiripentol.

**CDKL5 Encephalopathy.** Mutations in the human X linked cyclin–dipendent kinase like 5 (CDKL5), infantile spasms and Rett (RTT) like phenotype. Initially normal EEG and severe hypotonia. Aquired microcephaly and hand stereotypes. Identified with early infantile epileptic encephalopathy 2. Prognosis severe.

